# Nomenclature and heterogeneity: consequences for the use of mesenchymal stem cells in regenerative medicine

**DOI:** 10.2217/rme-2018-0145

**Published:** 2019-05-22

**Authors:** Alison Wilson, Andrew Webster, Paul Genever

**Affiliations:** 1 Department of Biology, University of York, York YO10 5DD, UK; 2 Science & Technology Studies Unit, Department of Sociology, University of York, York YO10 5DD, UK

**Keywords:** advanced therapy, clinical trial, heterogeneity, mesenchymal stem cells, mesenchymal stromal cells, MSC, nomenclature, regenerative medicine, therapeutic use

## Abstract

Mesenchymal stem cells (MSCs) are in development for many clinical indications, based both on ‘stem’ properties (tissue repair or regeneration) and on signaling repertoire (immunomodulatory and anti-inflammatory effects). Potential conflation of MSC properties with those of tissue-derived stromal cells presents difficulties in comparing study outcomes and represents a source of confusion in cell therapy development. Cultured MSCs demonstrate significant heterogeneity in clonogenicity and multi-lineage differentiation potential. However *in vivo* biology of MSCs includes native functions unrelated to regenerative medicine applications, so do nomenclature and heterogeneity matter? In this perspective we examine some consequences of the nomenclature debate and heterogeneity of MSCs. Regulatory expectations are considered, emphasizing that product development should prioritize detailed characterization of therapeutic cell populations for specific indications.

Variation is a fundamental concept in biology. While conservation of genes over evolutionary time spans allows for the preservation of essential processes common to all life it is variation that enables adaptation and survival. Within species, biological and behavioral traits exhibit a continuous spectrum of variation [1] which are likely to be based in part on variations in gene expression [2]. Even highly conserved RNA genes exhibit both species differences and variations in expression across different tissues [3].

Within a clonal population of cells, variations in gene expression between individual cells arise due to both extrinsic and intrinsic factors which determine the exact profile of gene expression and biological activities [4]. Since changes in signaling activity will impact upon the environment of other cells in the population, heterogeneity is inevitable even when the cells are genetically identical. Heterogeneity in cell communities may in fact be critical to many biological processes [5], but is generally not considered in the routine characterization of cell populations, where properties are frequently reported on an averaged basis. Although variation is inevitable, limitations in our ability to detect and control heterogeneity brings with it challenges for the production of cell therapies in which cells are the active substance in a medicinal product. Increasingly sophisticated techniques allow elucidation of expression profiles at the single cell level [6] which may provide insights useful for the optimization of cell culture for regenerative medicine products. Since one of the goals of medicinal product manufacture is consistency, can we reconcile variation at the individual cell level, for example as detected in RNA sequencing [7] or microfluidics [8], with the population-based measurements currently used to characterize cells for regenerative medicine? How closely should we seek to control cell phenotype and expression profile to achieve a therapeutic goal? Are there benefits of population heterogeneity for the therapeutic effects of the product?

The regulatory frameworks for medicinal products, which includes cell therapies, require developers to define and produce consistently a specific product which is controlled in terms of its quality attributes. Developers need to consider how to achieve routine manufacture of safe and efficacious cell therapies when the very nature of the starting material seems to undermine this objective.

Mesenchymal stem/stromal cells (MSCs) represent a significant fraction of the current efforts to develop cell-based treatments for a range of diseases. There are at present 98 clinical trials involving mesenchymal stem/stromal cells as the investigational medicinal product registered with the European clinical trials database EudraCT (Figure 1). The colony-forming fibroblastic adherent cell population originally described by Friedenstein *et al.* [9] have become the cell of choice for many regenerative medicine applications, and the literature expands daily.

**Figure 1.**
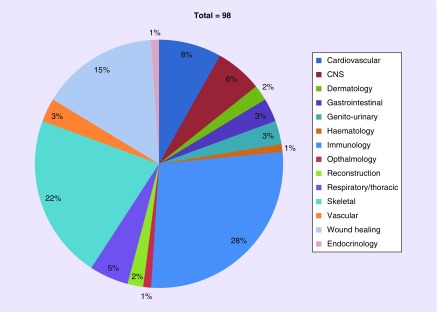
EU clinical trials involving ‘mesenchymal stem cell’. A total of 27 (28%) of the 98 mesenchymal stem cell clinical trials currently registered on EudraCT involve immunomodulatory properties of mesenchymal stem cell. A total of 22 (22%) are skeletal applications (bone, tendon repair, osteoarthritis), 15 (15%) address wound healing applications (skin ulcers, burns, fistulae). Cardiovascular (eight trials, 8%) and CNS (six trials, 6%) indications cover the majority of other trials. Source: EudraCT www.clinicaltrialsregister.eu (Accessed 3 November 2018).

In this perspective we consider the impact of biological heterogeneity on some of the regulatory requirements to which MSC-based therapies are subject, and discuss how these factors might impact upon the use of MSC in regenerative medicine.

## MSC nomenclature

One of the most challenging aspects of MSCs is the perennial debate over nomenclature: ‘stem’ versus ‘stromal’ and thus identity. Stem cells may be defined by two broad properties: the capacity for self-renewal and symmetric and asymmetric division, through which they produce lineage-committed progenitors which ultimately differentiate into tissue-specific cells [10]. Stem cell homing in response to specific cues results in formation of new functional tissue *in vivo* [11].

The term ‘mesenchymal stem cell’ originated with Caplan [12] following success in generating cartilage and bone tissue from *ex vivo* culture of embryonic chick mesenchymal tissue. Similar findings were obtained using cultured cells derived from the periosteum; the author did not examine other tissues but contended that a similar approach would be suitable to assess other mesenchymal tissues. This paper was one of the first to suggest the potential for use of *ex vivo* culture-expanded cells to produce replacement skeletal tissues as a therapy.

The literature abounds with descriptions apparently conflating bone marrow-derived *stem* cells, which combine demonstrated self-renewal with intrinsic skeletogenic differentiation potential, with *stromal* cells from a range of different tissue sources, both structural and nonstructural. A multiplicity of terms, each with its own implicit assumptions, has arisen, and despite repeated calls for clarity rooted in the specific biology of the cells, notably from the International Society for Cell and Gene Therapy (ISCT) [13] and others [14–17], many reports contribute to the confusion by failing to distinguish between true stem cells residing in the bone marrow and a variety of clonogenic stromal populations with varied characteristics.

The ISCT recommended a clear distinction between the bone marrow-derived self-renewing fraction with proven multi-potent differentiation *in vivo* (mesenchymal stem cells) and mesenchymal stromal cells from multiple tissues, shown to be multi-potent via *in vitro* differentiation assays [13]. Since the acronym ‘MSC’ was already embedded in the literature, the ISCT did not recommend a new term but rather emphasized the importance of definition of stem or stromal cell within studies. The use of the ‘MSC’ acronym is even more widely used now than in 2005, thus there is no attempt to redefine terms here, but rather to reiterate the need for meaningful descriptions of cell populations based on properties rather than expectations.

‘MSCs’ are described in the literature in broadly two ways: firstly specifically the rare cellular component of bone marrow, proven to be self-renewing, clonogenic and capable of producing skeletal tissues only, via *in vivo* serial transplantation [16,18]. This approach to derivation and characterization followed the paradigm used for hematopoietic stem cells, in which individual clonal populations have been evaluated by serial transplantation into recipient animals, thereby demonstrating both self-renewal and multipotency. Alternatively MSC are stromal progenitors found in multiple tissue types, which can be induced to differentiate *in vitro* into different lineages beyond skeletal tissues [19,20]. Much of this literature has to a large extent used a panel of surface markers, individually not necessarily specific for MSCs, and properties such as those proposed by the ISCT position statement [21] (Table 1) to characterise a wide range of cells from many different tissue sources.

**Table 1.** International Society for Cell and Gene Therapy criteria for identification of multipotent mesenchymal stromal cells.

Characteristic		Requirement
Plastic adherence		Adherent
Surface antigens	CD73, CD105, CD90 CD34, CD45, CD14 or CD11b, CD79α or CD19, HLA-DR	≥95% positive ≤2% positive
Differentiation potential *in vitro* to:		Osteocytes Chondrocytes Adipocytes

The ISCT criteria were intended to address the increasing difficulties in comparing outcomes from studies with cells isolated from different tissues and via different culture protocols. Although the authors stated that they were not intended to serve as release criteria for clinical applications, the ISCT criteria have become a *de facto* ‘standard’ for MSCs: many research papers, and also clinical trial applications [22] appear to rely on these criteria as being sufficient to characterise the population under investigation. However none of the parameters are specific to MSCs [23,24]. Although widely used in primary research and as a tool to confirm multipotentiality, the standard *in vitro* differentiation assays have been criticized for their lack of specificity and robustness [17].

A further use of the MSC acronym has been proposed, this time for Medicinal Signaling Cell [25–27] based on cells’ expression of trophic and immunomodulatory factors rather than differentiation capacity. Abandonment of the general MSC term and replacement with tissue-specific stromal cell descriptors has been recommended [17].

## MSCs *in vivo*

The existence of a nonhematopoietic stem cell within bone marrow was confirmed via a number of key studies, summarized by Bianco [18]: i*n vivo* transplantation of increasingly well-defined elements of the bone marrow showed that transplanted fragments of whole bone marrow induced formation of bone and hematopoietic microenvironment in heterotopic organoids. Transplantation of clonally derived populations located skeletal potential in individual progenitor cells. Eventually serial transplantation of individual putative bone marrow stem cells demonstrated that CD146 identifies an *in vivo* population (sub-endothelial adventitial reticular cells [ARC] in the walls of bone marrow sinusoids) and that selection by CD146 expression isolates a population including clonogenic, self-renewing multi-potent cells capable of forming both bone and hematopoiesis-supporting stroma upon transplantation.

MSCs are an integral component of the hematopoietic niche in bone marrow [28,29]. The composition of this niche and the role of MSCs within it has been investigated extensively over the last 10 years, with progress reviewed in, Hanoun and Frenette [30], Morrison and Scadden [31], Asada *et al.* [32]. Briefly, the nonhematopoietic, nonendothelial stem cell fraction within human bone marrow which is crucial for niche maintenance has been prospectively identified by expression of CXCL-12, (MCAM)/CD146 and expression of angiopoietin-1 [29], the pericyte marker NG2 and PDGF-β [19]. Single CD45^-^/CD146^+^ cells expanded from human bone marrow establish both hematopoietic tissue and bone organoids when transplanted ectopically [29], thus meeting expectations for a true stem cell. *In situ*, CD146 expression is limited to ARCs within bone marrow sinusoid walls; these cells are endothelial marker-negative (CD31/PECAM, CD133, VEFGR2, VE-cadherin) but express markers of pericyte (α-SMA, PDGFR-β, calponins 1 & 3) and mural cell origin (NG2) [29,33]. Low-affinity nerve growth factor receptor (CD271) is co-expressed with CD146 in perivascular locations, but absence of CD146 expression (lin^-^/CD271^+^/CD45^−^/CD146^−^) allows *in situ* localization of another population of MSCs to endosteum [34]. Recently Chan *et al.* [35] reported that a PDPN^+^/CD146^-^/CD73^+^/CD164^+^ phenotypic profile identifies a human skeletal stem cell (SSC) associated with growth plate rather than bone marrow, which is clonogenic *in vitro* and produces bone, cartilage and hematopoietic stroma *in vivo.* These findings mark a departure from the usual picture of bone marrow-derived MSC, in that adipogenic differentiation was not observed, and in contradiction to other studies, the SSCs lack CD146 expression which locates MSC in perivascular (sinusoidal) sites [29,34]. It is thus possible that the population identified by Chan *et al.* [35] represents a dedicated skeletal lineage independent of the marrow-derived populations investigated to date.

## MSCs *in vitro*

Bone marrow stromal cells, traditionally isolated from marrow via plastic adherence, form fibroblastic cell colonies (colony-forming units-fibroblastic or CFU-Fs) [9] which form individual colonies when seeded at clonal density [36]. Expansion of single colonies reveals a mixture of multipotent, uncommitted cells and lineage-committed progenitors [37–39]. However colony formation alone is insufficient to demonstrate stemness [16]. Multipotency and self-renewal can only be demonstrated at the single cell level, since nonclonal populations may contain multiple different committed progenitors which are selected for by the culture conditions, without the original population ever containing a true stem cell [14].

Álvarez-Viejo *et al.* [40] have highlighted the current absence of definitive identification criteria for MSC in fresh bone marrow aspirate and other tissue sources. Markers such as Stro-1, SSEA-4, CD146, CD271, CD49f (α-6 integrin), MSCA-1 and 3G5 may be valuable alone or in combination for both isolation/enrichment of MSC populations within cultures, and for selection of subsets with greater CFU-F and multipotency [40,41]. Many studies have investigated the surface marker expression profile of cultured MSC, which have been reviewed extensively by Mafi *et al.* [42], Calloni *et al.* [43], Kobolak *et al.* [44] and Samsonraj *et al.* [45].

## Heterogeneity of MSCs

Any culture of stromal cells isolated from primary tissue will be a heterogeneous mixture: for example, bone marrow aspirate contains a variety of hematopoietic cells, red blood cells and stromal cells including fat cells, endothelial cells, fibroblastic cells and marrow stem/progenitor cells [46]. The initial isolation procedure for MSCs frequently involves adherence to plastic. This characteristic, a key component of the ISCT’s identity criteria for multipotent MSCs, separates nonadherent hematopoietic stem cells from the adherent fraction that is assumed to be the ‘mesenchymal stem cell’ fraction. However fibroblasts have similar properties including plastic adherence [47] and proliferation to >50 population doublings before senescence [48].

Donor variation is well recognized as a fundamental source of variability in MSC populations, including in growth kinetics, and thus potential yields between donors and immunomodulatory capacity [49]. Donor age and gender impact both yield and immune-suppressive functions [50]. Interdonor variability may also differ depending on tissue source [51,52]. These variations will impact upon clinical and commercial development of MSC cell therapies, especially autologous therapies, with respect to defining the characteristics critical for required clinical effects.

Populations of MSC in culture will contain different proportions of true stem cells and differentiation-committed progenitors. Individual cells within a culture proliferate, differentiate and senesce at different rates, such that it cannot be accurate to represent a culture of bone marrow stromal cells as a homogeneous population of MSCs [16]. Cultures seeded at nonclonal densities will produce mixed populations of adherent cells, some of which arise from clonogenic cells but others from nonclonogenic cells, which will be limited in their growth potential. Cultures re-established from single clones contain clonogenic self-renewing stem cells but these cultures become heterogeneous, reflecting the fundamental heterogeneity of the starting material [29,37].

Single colony-derived bone marrow stromal cells vary in their potential to induce bone formation *in vivo*, compared with polyclonal populations, which invariably form bone upon transplantation [53]. *In vitro* differentiation potential is likewise variable between individual clones: in one study >20% of clonally-derived human stromal cell strains showed tri-lineage differentiation potential to all three osteogenic, chondrogenic and adipogenic (OAC) lineages *in vitro*, with the majority being osteogenic-chondrogenic (OC) bi-potent clones [54]. This study reported absence of clones with OA or CA bipotential, and chondrogenic-only, adipogenic-only and nullipotent clones. Similar work produced all possible combinations of tri-, bi-, uni- and nulli-potent clones [39]; these differences were ascribed to experimental and culturing differences, which in itself highlights the difficulty of comparing outcomes across studies. These studies indicate a hierarchical specification resulting in heterogeneous functionality within MSC populations [55].

Populations expanded from single colonies of human bone marrow stromal cells from a single donor show wide variation in differentiation potential following *in vivo* transplantation: 67% bone-forming but only 12.5% forming bone and hematopoietic tissue, and around 20% forming only fibrous tissue [56]. Multi-potency appears related to other stem-like properties: clones showing differentiation potential to all three lineages are likely to be those with higher colony-forming capacity, faster doubling times and slower progression to senescence *in vitro* than those with uni- or bi-potency [57]. These studies all support the prevailing view that multipotent stem cells represent only a small fraction of the total nucleated bone marrow stromal cell population, and that clonogenicity alone is not indicative of stemness. Colony-forming assays in isolation overestimate the proportion of stem cells in a sample of bone marrow or other material, since committed osteoprogenitors are clonogenic but uni-potent [58]. *Ex vivo* markers of osteoblastic phenotype (e.g., ALP) were not predictive of the *in vivo* bone-forming capacity. Therefore, it is of a great interest to define *ex vivo* molecular markers that are better at predicting the *in vivo* bone-formation capacity of BMSCs.

The preceding studies used nonimmortalized bone marrow stromal cells in extended culture, which invariably results in loss of differentiation potential [54]. Immortalization of MSCs by retroviral transduction with human telomerase reverse transcriptase (hTERT) complementary DNA bypasses culture-induced senescence and maintains proliferative and multi-lineage differentiation capacity over >260 population doublings [59]. Availability of practically inexhaustible stocks of consistent MSCs allows for detailed analyses of the potential of populations derived from single cells. MSCs from a single donor, immortalised via lentiviral transduction with hTERT, produce a range of clones demonstrating both multi-potent differentiation capability and nullipotency [60]. Global gene expression arrays identified distinct phenotypes, with multipotent clones showing upregulation of a range of vascular development and growth genes, and an inflammatory gene profile including IFN-γ, TNF-α and IL-7 in the poorly differentiating clones. The inflammatory clones expressed CD317, and selection by CD317 identified a small fraction (1–3%) with high IL-7 expression within primary stromal cell culture, suggesting that these clones represent a subset within primary stromal cell populations. Similarly Elsafadi *et al.* [61] reported on two clones from hTERT-MSC that displayed fundamentally different phenotypes: one expressed high levels of osteogenic markers (alkaline phosphatase and CD146), bone and skeletal muscle-related genes, and differentiated to bone, fat and cartilage *in* *vitro*; the other expressed increased immunomodulatory and immune defence genes and showed greatly reduced tri-lineage differentiation potential. Of note, clones from both studies all expressed a range of ‘expected’ MSC markers including CD29, CD44, CD63, CD73, CD90, CD105 and CD166 despite such large differences in differentiation potential.

The use of immortalization to facilitate reproducible studies on consistent cells is a valuable research tool that allows exploration of the inherent heterogeneity of MSCs but such cell lines may not reflect the natural organization or characteristics of bone marrow stromal cells either *in vivo* or in short-term nontransformed culture, the latter being more likely to be used for production of cell therapy medicines. The preceding studies illustrate the difficulty in producing a consistent population of cells for therapeutic use. Even with tissue from a single donor, controlled culture conditions and expansion from a single cell, each clone produces a distinct population with widely different morphology, growth kinetics, gene expression profile and functional protein expression.

## Issues for regenerative medicine

### MSC in regenerative medicine: a range of perceptions

Reporting of the isolation of stromal cells possessing multilineage differentiation capacity from a wide range of tissues including adipose, placenta, umbilical cord (UC) and dental pulp has led to a situation in which attributes observed *in vivo* from bone marrow-derived MSC have been extrapolated to make assumptions about cultured cells. These assumptions have apparently been the basis of a rationale for clinical application of expanded MSC in a variety of therapeutic indications. These applications reflect expectations based on the current understanding of the behavior of MSCs *in vitro*, and suggest an assumption that properties exhibited in a culture environment will necessarily be maintained upon administration to a patient.

**Figure 2.**
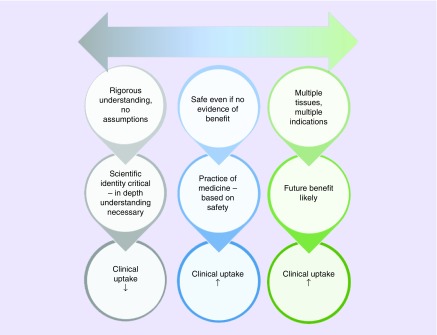
Spectrum of approaches to mesenchymal stem cells in regenerative medicine. Literature concerning use of mesenchymal stem cells in clinical applications appears to represent a spectrum of opinions: at one end of the spectrum strong support for exploring a vast range of therapeutic indications using cells from a range of tissues, and at the other a more cautious, strictly evidentiary approach that emphasizes the importance of detailed empirical support for all likely mechanisms and avoidance of any assumptions whatsoever regarding anticipation of clinical benefit. The rate of clinical update may be supported by a more exploratory approach based on assumptions concerning ‘generic’ mesenchymal stem cell properties.

The apparent acceptance that all tissue sources contain stem cell populations comparable to those seen in bone marrow stroma has led to a noticeable divide in published views of the use of MSC in clinical development (Figure 2): at one end of the spectrum there is strong support for exploring a vast range of therapeutic indications using cells from a range of tissues, and at the other a more cautious, strictly evidentiary approach that emphasises the importance of detailed empirical support for all likely mechanisms and avoidance of any assumptions whatsoever regarding anticipation of clinical benefit. Somewhere in the middle, the ever-increasing pool of clinical reports may encourage exploratory use based on the lack of significant adverse events being reported, although in isolation this should not be considered a reliable indicator of patient safety.

The literature clearly highlights the extensive variation among populations of MSCs whether arising from tissue source, culture conditions or population doublings, and one of the most important aspects with relevance to regenerative medicine is the extent to which a population of MSCs derived from a single donation/tissue can vary. It will be important, and also challenging, to elucidate the profiles of subsets most promising for different indications, which implies identification of subsets with relevant gene/protein expression for the intended function and ability to isolate these subsets based on accessible epitopes.

### Differences between tissue sources

The ability to culture such colonies of stromal cells from many different tissues has contributed to the expectation that multiple sources contain cell populations with analogous properties to bone marrow-derived stem cells [19,20]. However differences between tissue sources are apparent: although absence of CD34 expression is stipulated in the ISCT minimal identity criteria for cultured MSCs [21], CD34 expression is recommended for fresh MSCs within stromal vascular fraction and is noted as an ‘unstable primary marker of cultured adipose-derived stromal/stem cells’ [62]. Although, presumably because of the nonspecificity of the ISCT marker panel, expanded stromal cells from many tissues meet the minimal criteria for MSC identity, differences in gene expression and differentiation potential between tissue sources are reported [52,63–65]. Stromal cells from non-marrow sources including adipose, UC and menstrual blood, have been shown to express different surface marker profiles [63,66], whereas synovial membrane-derived stromal cells appear phenotypically closer to bone marrow-derived MSCs [67]. Perinatal tissues represent an accessible source of cells for regenerative medicine without the necessity for invasive harvesting procedures. Whilst generally reflecting the expected MSC surface markers, functional differences between sources are apparent. MSCs from UC blood show considerable heterogeneity in terms of expansion and immunomodulatory capacity [68]. There are reports that UC-derived MSCs (UC-MSCs) have greater expansion capacity, greater osteogenic and adipogenic potential, and higher CD146 expression than bone marrow MSCs [63,69]. MSCs from different layers of the placenta show variation in proliferation and differentiation capacity [70], and MSCs from amnion also show variable differentiation potential and high inter-donor variability compared with UC-MSCs [52].

The developmental origins of MSC may include neural crest [71]. Further heterogeneity of stromal cell populations from bone marrow, adipose and skin is evidenced by the presence of neural crest-derived stem cells [72,73] within the population expressing expected MSC markers CD73, CD90 and CD105.

**Figure 3.**
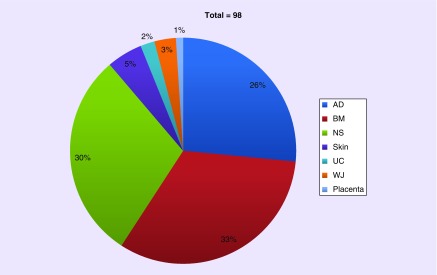
Tissue sources in EU mesenchymal stromal cells clinical trials. From the 98 clinical trials involving mesenchymal stromal cell as the investigational medicinal product currently registered on EudraCT, 32 (33%) stated the source of mesenchymal stromal cell as BM, 25 (26%) utilized AD and 29 (30%) did not specify the NS in the primary record or the Competent Authority application form. Skin, UC, W and placenta were also mentioned as source tissues. Source: EudraCT www.clinicaltrialsregister.eu (Accessed 3 November 2018). AD: Adipose tissue; BM: Bone marrow; NS: Source tissue; UC: Umbilical cord; WJ: Wharton's jelly.

The explosive growth of the MSC cell therapy industry has been based, in part, on the expectation of tissue/source equivalence, with 26% of current EU clinical trials using adipose-derived MSCs, and 30% not stating the tissue origin in the publicly accessible trial details on the EU clinical trial register EudraCT (Figure 3). Although tissue source will have been disclosed to the regulatory authorities, it is interesting that the trial sponsors did not apparently consider it to be a significant detail in the main application forms for the clinical trial authorization.

### The potency assay: linking identity & variability to regulatory expectations

Medicinal products, including cell therapies, are regulated on the basis of their intended therapeutic indication. That is, the applicant for a clinical trial or marketing authorization has to define what condition is to be treated or prevented, or what clinical effect the medicinal product is intended to achieve. Early in product development, there may be only prior literature, or hints from primary research, to guide identification of mechanisms that could deliver potentially useful clinical effects. These clues must ultimately be crystallized into a package of data that identifies the active moiety (chemical substance, biological molecule or cellular component) and demonstrates its safety and effectiveness in the proposed clinical indication. Elucidation of relevant mechanisms of action is thus a key aspect of development of cellular therapies. While it may be almost impossible to identify all possible mechanisms, an understanding of the major properties likely to result in the intended biological activity is essential.

For the medicinal product to be licensed, allowing it to become accessible to patients on a routine basis, regulatory requirements must be met. A critical aspect of development of all biological medicines is the requirement for a potency assay: one or more assays capable of confirming that the batch of product meets established specifications for relevant biological activity when compared against a reference standard or performance criterion, thus ensuring consistency of production [74,75]. Potency assays are expected to be correlated with clinical performance, allowing confirmation that each batch has the same biological functionality as those tested in clinical trials. Since the potency assay must relate to a biological property relevant for the intended indication, quantitative measures based on understanding of the specific mechanisms of action are required. The challenges of identifying relevant properties for cell therapies are significant because, unlike conventional medicinal products, the administered cells are likely to interact in a complex and potentially unpredictable manner with the recipient’s tissues and physiological mechanisms.

Consideration of the requirement for a potency assay, or more likely a combination of complementary assays, highlights the necessity of understanding the broad mechanisms of action of the product. Immunomodulatory properties of MSC have been studied extensively in *in vitro* and *in vivo* assays [76–78]. Although often characterised by suppression of T-cell proliferation induced by mixed lymphocyte reactions or other pro-inflammatory stimuli, the specific mechanisms by which MSCs achieve these effects are complex and multimodal [79]. Recent ISCT publications discussed approaches to developing potency assays in immunomodulatory applications [80,81]. Table 2 illustrates a range of properties of MSCs which may be suitable for development as potential potency assays for mesenchymal stem/stromal cell therapies.

**Table 2.** Properties of mesenchymal stromal cells with potential for potency assay development.

Indication	Properties relevant to potency assay development	Ref.
Multiple organ dysfunction syndrome	IL-10 release	[82]
Graft-vs-host disease	TNF-R1 expression	[83]
Multiple immune/inflammatory conditions	T-cell proliferation suppression	[49]
	CD4+ T-cell proliferation suppression	[84]
	TNF-α inhibition	[85]
Corneal damage from chemical insult	TNF-α stimulated gene/protein 6 (TSG-6) expression	[86]
Acute myocardial infarction	*In vitro* tubule formation (CXCL5, IL-8, VEGF expression)	[87]
Cartilage repair	Receptor tyrosine kinase‐like orphan receptor 2 (ROR2) expression	[88]

For cellular therapies and in particular those intended for tissue repair/regeneration, there are likely to be a range of mechanisms involving secretion of trophic support molecules [26,89–91]. The clinical exploration of MSCs for neurological conditions including multiple sclerosis and stroke has been justified based on such mechanisms [92–94]. *In situ* differentiation into site-specific tissue for repair of tissues/organs, once a cornerstone of the MSC treatment paradigm, is increasingly rejected as evidence of lack of engraftment and persistence following intravenous or local injection accumulates, pointing to paracrine effects rather than replacement with differentiated tissue *de novo* for nonskeletal indications [89,95,96]. Inherent donor-related variability in immunosuppressive activity may account in part for inconsistent clinical trial outcomes [97]. The MSC secretome and thus cells’ paracrine activity is profoundly impacted by microenvironment [98]. Immunomodulatory activity in particular requires a pro-inflammatory environment to prime MSCs [99] thus preconditioning of MSCs with cytokines may increase expression of potentially therapeutic molecules [100,101]. Priming MSCs with Toll-like receptor (TLR)-3 agonists induces an immunosuppressive phenotype [102]. Aside from paracrine mechanisms of action, priming of different TLR family members may impact upon differentiation potential [103,104], although the therapeutic value of this observation is unclear given that site-specific differentiation of MSCs in bone and cartilage injury has yet to be definitively confirmed in clinical trials.

For many regenerative applications, stem properties (self-renewal, multipotency) may therefore not be relevant at all. In this vein, the concept of MSC as ‘medicinal signaling cells’ arises [25,27]. Production and delivery of therapeutic molecules via MSC-derived exosomes, intracellular nanoparticles involved in intercellular signaling and release of lipids, proteins and nucleic acids, is mooted as a possible alternative to the use of MSC themselves as the therapeutic agent [105]. The potential of MSC-derived exosomes is under exploration in numerous areas including myocardial infarction [106], osteoarthritis [107] fracture healing [108] and neurodegenerative disease [109]. Composition and activity varies in exosomes from different tissues [110,111]. Exosome-based therapy may avoid some potential risks of cell administration, but challenges around mechanism of action, production at scale and consistency will need to be addressed in the same way as for MSC-based therapies [112].

**Figure 4.**
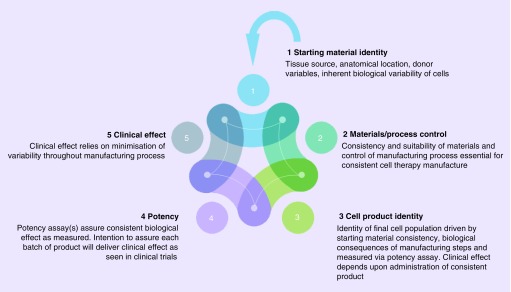
Identity as an integral part of cell therapy manufacture. Each aspect of the manufacture of consistent and effective cell therapies is linked: heterogeneity of the starting material (tissue/cell source) is a fundamental source of variability which impacts upon the overall ability of the process to deliver an effective product with consistent relevant biological functionality equivalent to that assessed in clinical trials.

With a vast range of potential molecules, pathways, networks and interactions that could contribute to clinical efficacy of a MSC-based cell therapy, assessment of the means by which it achieves its effects becomes incredibly challenging. Fortunately regulators in the EU and the US do not expect fully developed potency assays as a condition of entry into clinical trials in human subjects; however a rationale to underpin the choice of indication and some evidence that the cell-based therapy can deliver relevant effects will be required before human trials begin, usually in the form of nonclinical pharmacology studies. Given the complexity of the potency issue, it is inevitable that there is a link back to identity of the cell population being developed. The identity profile needs to be defined during development, such that the impact of materials used for production, the control and consistency of processes employed can be assessed to ensure product of a consistent and relevant biological functionality can be generated. This in turn supports the production of consistent batches of cell product for the intended clinical effect: all are integrally linked (Figure 4). Thus understanding of the identity of the population is critical, and investigation of the relevant phenotypic and functional attributes is a fundamental aspect of cell therapy development. Clearly the heterogeneity associated with MSC populations creates additional complexity in terms of the conventional requirement to define the ‘drug substance'.

A more defined phenotype capable of predicting a required biological function *in vivo* should facilitate production and clinical evaluation of cell therapies [113]. However a key challenge in therapeutic application of MSCs appears to be that the surface markers commonly associated with *in vitro* functionality are not necessarily related to the corresponding activity *in vivo.* Global gene expression analysis may allow the elucidation of relationships between phenotype and function by highlighting possible relationships that are not immediately apparent [56]. However, large differences in expression (>tenfold) can be seen in cell strains with the same differentiation potential, underlining the difficulties in correlating gene expression with *in vivo* function.

## Impact of heterogeneity on cell therapy manufacture

MSCs are a major candidate for a wide range of potential therapeutic applications. Although the actual cell numbers required to treat an individual patient may vary with indication, it is certain that the overall numbers required to produce commercially and clinically viable products will necessitate effective expansion strategies. However the expansion of MSCs in adherent culture is known to result in slowing and eventual loss of proliferation [114] and loss of multi-lineage differentiation potential [115,116]. Possible strategies for countering these effects may include culture in hypoxic conditions, which affects MSC proliferation, differentiation capacity, migration and metabolism [117]. Hypoxic conditions can result in lower intracellular concentrations of reactive oxygen species (ROS), which are implicated in multiple adverse mechanisms during cell expansion (e.g., telomere shortening, chromosomal damage) [118].

The current challenges in identification of MSCs with true stem potential means that the expanded cells administered to a patient may comprise a heterogeneous population identified only by plastic adherence and the expression of a few nonspecific surface markers. This is of particular importance in early clinical trials, in which the supporting functional evidence generated in small animal models may have been achieved with much smaller cell numbers produced via fewer population doublings: a less expanded population of MSCs will likely represent a different population with differing proliferation and differentiation capacity. Differences in administered populations may result in failure and rejection of promising therapies when results in animal studies are not replicated in early clinical trials. Although difficult to assess this directly, it is certainly the case that many successful studies in animals do not translate/have not yet translated to positive results in the clinic. Whilst regulators do not currently require cell-based products to be absolutely pure, and in any case there would be significant challenges in defining what this means in practice, certain regenerative medicine applications may benefit from use of a clonal population rather than a heterogeneous material expanded from multiple primary cells [119].

Studies of culture methods intended to increase yields of MSCs for clinical use tend to quantify output by characterizing the expanded populations in terms of phenotype, plus occasionally morphology and immunosuppressive activity, for example, Gottipamula *et al.* [120], Haack-Sorensen *et al.* [121]. Similarly efforts to create biobanks of MSC have been assessed on the basis of ISCT or similar criteria alone [122]. These are entirely reasonable approaches for evaluation of a manufacturing process, but for the reasons already discussed, these criteria do not adequately identify the stem/progenitor content of the population and may thus tend to over-estimate the relevance of the output cells for some clinical applications.

## Future perspective

Different populations showing multi-potentiality *in vitro* can be isolated from many stromal tissues. The presence of true stem cells has been demonstrated in bone marrow [29] and in fetal and adult bone [35], but ‘stemness’ appears to be assumed in other tissue sources. Identification of cells as stem or multipotent stromal is a crucial distinction from the biological perspective and it should be a priority to define clearly the terms and assumptions in this regard in study publications. But how important is this for regulatory aspects in relation to regenerative medicine? If a population only contains a small proportion of true stem cells as defined in specifications, is this important? It is clear that the cultured MSCs embraced by the regenerative medicine community are not equivalent in all respects to the native population residing in the perivascular/sinusoidal hematopoietic niche. They do not have, indeed are not required to have, the same functions, in that they are not intended to support the HSC niche. Similarly, the production of new bone in natural skeletal replenishment or repair, orchestrated by a specific and controlled sequence of physiological signals, is not likely to be recapitulated during administration of *ex vivo* expanded MSCs. Regulatory authorities recognize the distinction between the native functions of cells and their potential uses in medicinal products. The cell therapy regulations in both the EU and the US make a distinction between cells intended to perform the same intended function as native cells and those for which the intended clinical purpose of the cells is different to that which the cells would normally perform in the body, with this so-called ‘nonhomologous use’ being regulated by medicines/biologics legislation (Box 1).

Box 1.  **European Union.**

- Regulation (EC) No 1394/2007 Article 2.1 (b) ‘Tissue engineered product’ means a product that:
  – contains or consists of engineered cells or tissues
- 2.1 (c) Cells or tissues shall be considered ‘engineered’ if they fulfill at least one of the following conditions:
  – the cells or tissues have been subject to substantial manipulation, so that biological characteristics, physiological functions or structural properties relevant for the intended regeneration, repair or replacement are achieved. The manipulations listed in Annex I, in particular, shall not be considered as substantial manipulations
  – the cells or tissues are not intended to be used for the same essential function or functions in the recipient as in the donor
- Directive 2001/83/EC Annex Part IV 2.2.(a): Somatic cell therapy medicinal product means a biological medicinal product which has the following characteristics: (a) contains or consists of cells or tissues that have been subject to substantial manipulation so that biological characteristics, physiological functions or structural properties relevant for the intended clinical use have been altered, ***or of cells or tissues that are not intended to be used for the same essential function(s) in the recipient and the donor;***

**USA**

- 21 CFR 1271.10
- a) An HCT/P (human cells, tissues and cellular and tissue-based product) is regulated solely under section 361 of the PHS Act and the regulations in this part if it meets all of the following criteria:
- …(2) The HCT/P is intended for homologous use only, as reflected by the labeling, advertising, or other indications of the manufacturer’s objective intent;

The rigor applied in primary research to further elucidating the locations, properties and functions of individual sets of bone marrow stem and stromal cells, and stromal cells from other tissues, is essential to help inform selection of appropriate populations for regenerative medicine applications. There is abundant evidence that stromal cells from different tissues exhibit differences in marker profiles, gene expression patterns and propensity to differentiate into particular cell types. Inherent heterogeneity of cell populations makes characterization challenging, but developers of regenerative medicines should take into account the basic biological attributes of their chosen cell type, perhaps considering the optimum tissue source and desired functionality based on a combination of fundamental biology and understanding of the impact of processing conditions during cell expansion.

Developers of MSC-based therapies need to be cautious in their assumptions about the identity and relevant mechanisms of action attributed to their cell population. The expression of a range of nonspecific surface antigen markers is to be expected for MSCs; in order to be relevant for regulatory identity requirements, developers should seek to identify combinations of markers more specific to the cell population produced in their particular manufacturing process. The ability of a specific cell population to deliver particular biological functionality must be explored in the context of the intended indication, and not by application of a generic *in vitro* differentiation assay that may have little or no specific relevance to that indication.

We should be mindful, however, not to paralyze the field of regenerative medicine with ambitious goals that may hinder valuable clinical progress: a balance between detailed understanding of native biology and practical analysis of the cell population under development is essential. It is important to emphasize that different stakeholders will have different interests and objectives: research scientists seek elucidation of the biology of cells within their native environment; regulators require that the specific cell population, in other words, the ‘drug substance’ for clinical application is characterized, and the cell therapy community could benefit from a standard set of criteria that may be helpful in providing a baseline for comparison of results. Does it matter what we call these cells when each clinical trial application requires individual identity, cellular composition and relevant potency criteria for the cells and process under consideration for a specific indication? From a purely regulatory perspective, probably not, but in order to allow for meaningful comparisons during research we should seek clarity of terminology and descriptions, avoiding universal attribution of properties elucidated under specific circumstances.

As the clinical use of MSCs increases, it would be of value to the research community to share key data. For example, publicly accessible databases such as the Stemformatics stem cell project [123] allow submission and sharing of gene expression and pathway data, enabling researchers to compare their data to others. Single cell RNA sequencing can characterise differences in the differentiation and immunomodulatory potential of MSCs at the single cell level [124]. Developers of MSC-based products may benefit from more comprehensive characterization data as the number of batches of cells increases: compilation and analysis of RNAseq data for cells used in clinical trials may eventually yield valuable insights in terms of the clinical consequences of heterogeneity of MSCs.

**Executive summary**

**Background**

- Variation is a fundamental concept in biology.
- Heterogeneity arises in clonal cell populations.
- Potential challenges for the regulatory framework because of mesenchymal stromal cell (MSC) heterogeneity.
- Clinical trials in the EU are exploring the use of MSCs in a wide range of different therapeutic applications.

**MSC nomenclature**

- Stem or stromal? Are the two terms conflated in the MSC literature?
- Definitions and additional ‘MSC’ acronyms, and the use of ‘standard’ identification criteria for cultured MSCs.

**MSCs *in vivo***

- Brief history of the identification and functions of MSCs within the hematopoietic niche.
- Phenotypic identification of a putative human skeletal stem cell.

**MSCs *in vitro***

- Identification of colony-forming units – fibroblastic within bone marrow stroma.
- Isolation and enrichment by cell surface markers.

**Heterogeneity of MSCs**

- Impact of donor age, gender and tissue source.
- Colonies form a heterogeneous mix of cells with varying self-renewal capacity and multipotentiality, and not a population of ‘stem’ cells.
- Cultures expanded from single colonies demonstrate extensive heterogeneity both within and between cultures.
- Single clones from immortalized MSC cell lines show profoundly different gene expression profiles and differentiation capacity.

**Issues for regenerative medicine**

- Perceptions of MSC: a spectrum of approaches to their use in regenerative medicine.
- Equivalence of tissue sources.
- The potency assay – linking identity and variability to regulatory expectations.
- Impact of heterogeneity on cell therapy product manufacture.
